# Brevilin A Isolated from *Centipeda minima* Induces Apoptosis in Human Gastric Cancer Cells via an Extrinsic Apoptotic Signaling Pathway

**DOI:** 10.3390/plants11131658

**Published:** 2022-06-23

**Authors:** Dahae Lee, Hee Jae Kwak, Byoung Ha Kim, Dong-Wook Kim, Hyun Young Kim, Seung Hyun Kim, Ki Sung Kang

**Affiliations:** 1 College of Korean Medicine, Gachon University, Seongnam 13120, Korea; pjsldh@gachon.ac.kr; 2 Yonsei Institute of Pharmaceutical Sciences, College of Pharmacy, Yonsei University, Incheon 21983, Korea; moon3685@naver.com; 3 D. Nature Co., Ltd., Seongnam 13174, Korea; mot37@d-nature.co.kr; 4 College of Pharmacy, Wonkwang University, Iksan 54538, Korea; pharmengin1@wku.ac.kr; 5 Department of Food Science and Nutrition, Gyeongsang National University, Jinju 52725, Korea; hyunyoung.kim@gnu.ac.kr

**Keywords:** *Centipeda minima*, brevilin A, network pharmacology, caspase-8, extrinsic apoptosis

## Abstract

Brevilin A, which has anticancer activities against a range of cancers, is an abundant constituent of the medicinal herb *Centipeda minima* (L.) A. Braun & Asch, which has also been reported to have anticancer activity against breast cancer cells. However, the anticancer activities of *C. minima* and brevilin A against human gastric cancer have yet to be reported. In this study, we aimed to evaluate the cytotoxicity and molecular basis underlying the anticancer activities of extracts of *C. minima* (CMX) and brevilin A against human gastric cancer (AGS) cells. We deduced the potential targets and mechanisms underlying the anticancer activity of brevilin A based on a network pharmacology approach. CCND1, CDK4, and BCL2L1 were identified as the key anticancer genes targeted by brevilin A. Cytotoxicity analyses revealed that CMX and brevilin A reduced the viability of AGS cells to levels below 50% (9.73 ± 1.29 µg/mL and 54.69 ± 1.38 μM, respectively). Furthermore, Hoechst 33342, annexin V, and propidium iodide staining and western blot analyses revealed that CMX and brevilin A promoted a significant induction of apoptotic cell death by upregulating the expression of cleaved caspase-8 and cleaved caspase-3 and reducing the ratio of Bax to Bcl-2, which is partially consistent with the findings of our network pharmacology analysis. Collectively, our observations indicate that CMX and brevilin A are novel sources of herbal medicine with potential utility as effective agents for the treatment of gastric cancer.

## 1. Introduction

Gastric cancer is a serious public health concern, the incidence of which is associated with age, stomach disease, *Helicobacter pylori* infection, and behavioral risk factors, such as those related to diet, alcohol consumption, and exercise. Although the development of endoscopic examinations has significantly increased the rate of the early detection of gastric cancer and timely surgical resection can yield good results, the high metastasis rate of this cancer continues to reduce long-term survival rates. Consequently, as an alternative, there has been considerable recent interest in developing novel therapeutic approaches. Given that conventional chemotherapeutic anticancer agents are often cytotoxic, not only to tumor cells but also to normal cells, novel natural phytochemicals have been proposed as attractive alternatives to these chemical agents. It is thus particularly desirable to evaluate the properties of natural phytochemicals with a view toward developing less toxic and more effective anticancer agents.

*Centipeda minima* (L.) A. Braun & Asch. is a medicinal herb widely distributed in East and Southeast Asia that has been used in the treatment of asthma, malaria, headache, and nasal allergies. It has also been extensively studied for its antibacterial, antioxidant, anticancer, neuroprotective, and hair growth stimulation effects. The findings of recent studies have confirmed that extracts of *C. minima* (CMX) have an anticancer effect against human breast cancer and nasopharyngeal carcinoma cells [1,2]. In addition, it has recently been reported that certain phytochemical constituents of CMX have potential utility for the development of anticancer agents [3]. For example, arnicolide D isolated from CMX has been demonstrated to have anticancer effects against human nasopharyngeal carcinoma cells [4]. Previous studies have also reported that brevilin A, of which CMX is an abundant source, shows anticancer effects on a range of different cancer cell lines, including those of lung, breast, liver, and colon cancers and melanoma [5,6,7]. Brevilin A is a natural sesquiterpene lactone that has been established to have a range of pharmacological effects, including anti-influenza viral [8], antiprotozoal [9], and anti-inflammatory [10] activities. We reported that CMX contains arnicolide D, arnicolide C, and microhelenin C along with brevlin A [11]. However, to the best of our knowledge, there have to date been no studies that have evaluated the anticancer activities of CMX and brevilin A with respect to gastric cancer or investigated its molecular mechanisms. 

Network pharmacology is a comparatively new approach in drug discovery studies that integrates bioinformatics, poly-pharmacology, and network analysis [12]. Its concept of “multi drug–multi target” is consistent with the characteristics of natural products that consist of numerous compounds. Recent studies have used network pharmacology to deduce the bioactive compounds and pharmacological mechanisms of a number of natural products. For example, in our previous study, we characterized the anti-adipogenic effects of two compounds derived from natural products using a combination of network pharmacology and molecular biological experiments [13]. In addition, the mechanisms underlying the previously reported blood-enriching effect of *Salvia miltiorrhiza* have been analyzed using a network pharmacology approach [14]. Accordingly, network pharmacology offers a new pharmacological perspective for identifying the active compounds, potential targets, and mechanisms of action of natural products. In this study, we applied a network pharmacology approach to predict the potential targets and mechanisms associated with the anticancer activity of brevilin A, and complemented this analysis with an investigation of the anticancer activities of CMX and brevilin A on gastric cancer and the underlying molecular mechanisms.

## 2. Results and Discussion

### 2.1. Network Pharmacology Analysis

#### 2.1.1. Prediction of Targets and Screening of Potential Targets

**Figure 1:**
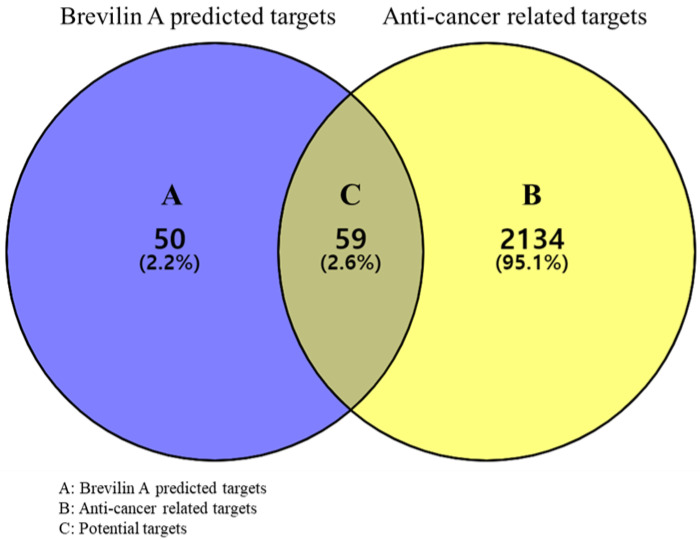
A Venn diagram of brevilin A predicted targets and anticancer-related targets.

In total, we obtained 109 predicted targets of brevilin A using the SwissTargetPredcition database and 2193 anticancer-related targets were acquired from the GeneCards database. Among these, we identified 59 targets commonly identified using both databases, which were selected as potential targets (Figure 1), the details of which are listed in Table 1.

#### 2.1.2. Construction and Analysis of Protein–Protein Interaction Networks of Potential and Key Targets

**Figure 2:**
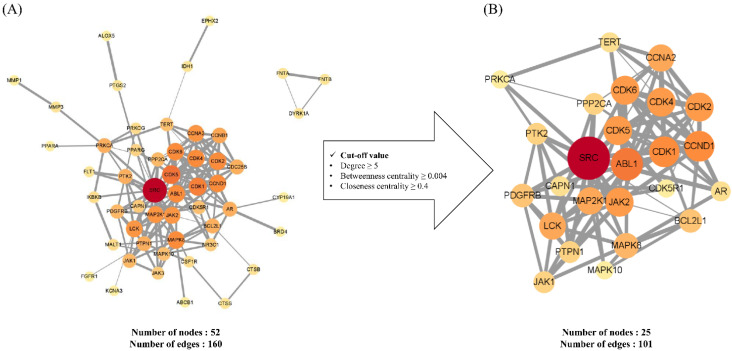
Protein–protein interaction (PPI) networks: (**A**) A PPI network of potential targets; (**B**) A PPI network of the key targets. The size and the red hue of a node denote its significance within the network.

PPI networks were established to gain a more in-depth understanding of the relationship among the target genes. Initially, all potential targets were input into the STRING database and the resultant data were subsequently subjected to Cytoscape visualization and analysis. Network analysis was performed based on the three parameters, degree (degree centrality), betweenness centrality, and closeness centrality, which were used to determine the importance of the different nodes within the complex network. The degree is a simple centrality measurement index that can be used to calculate the number of connections of a given node, with a high degree value indicating the relatively high importance of a node within the network [15]. Betweenness centrality provides an indication of the number of shortest inter-node paths, which thereby contributes to identifying those nodes that play a pivotal bridging role in the network [16]. Closeness centrality is defined as the length of the shortest path between two nodes, which indicates that a node with high closeness centrality has close relationships with numerous other nodes [17]. As shown in Figure 2, PPI networks are constructed with nodes that represent the target genes and edges that indicate the links between target genes. The size and color of a node denote degree intensity. Thus, the higher the degree of the node, the larger the node and the closer the color from yellow to red. The width of the edge indicates the grade of the correlation between the targets, and thus the larger the combined score, the higher is the binding degree between nodes and the thicker the edge.

Figure 2A depicts the PPI network of potential targets consisting of 52 nodes and 160 edges. To identify the key targets among the potential targets, we set the following cut-off values: degree ≥ 5, betweenness centrality ≥ 0.004, and closeness centrality ≥ 0.4; among the assessed targets, 25 satisfied all these cut-off values, and accordingly, the PPI network of key targets consisted of 25 nodes and 101 edges (Figure 2B). Among these, SRC (SRC proto-oncogene tyrosine-protein kinase SRC), ALB1 (calbindin 1), CDK1 (cyclin-dependent kinase 1), CCND1 (cyclin D1), CDK2 (cyclin-dependent kinase 2), CDK6 (cyclin-dependent kinase 6), CDK4 (cyclin-dependent kinase 4), CDK5 (cyclin-dependent kinase 5), and JAK2 (Janus kinase 2) were assigned values of 10 degrees or greater (Table 2). Of these, SRC, for which we obtained the highest degree node, is known as a key regulator associated with multiple aspects of cancer development, progression, adhesion, and invasion in numerous types of tumor and has previously been highlighted as an attractive target for anticancer therapeutic strategies [18,19]. Among the other identified genes, ALB1, CDKs, CCND1, and JAK2, have been established to play key roles in cell survival, adhesion, and invasion in tumors [20,21,22,23]. Accordingly, these finding would tend to indicate that these key targets could be significantly associated with the anticancer activity of brevilin A.

#### 2.1.3. KEGG Pathway Enrichment Analysis of Key Targets

**Figure 3:**
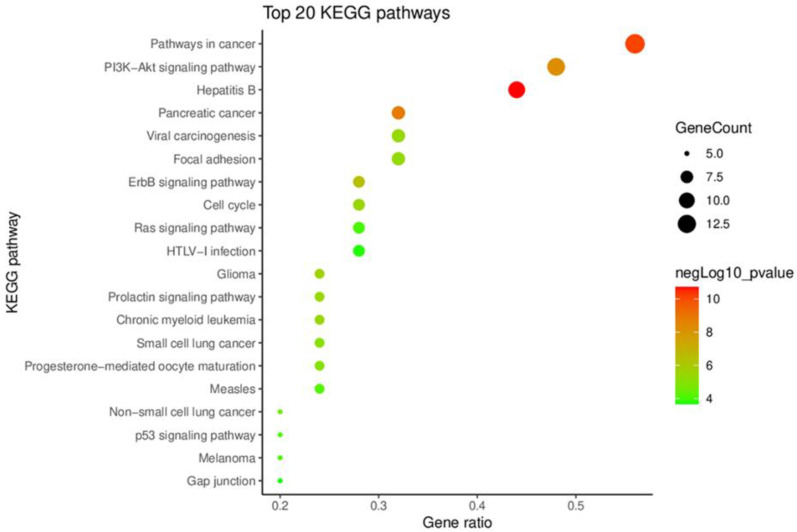
A bubble diagram of the top 20 pathways identified based on KEGG pathway enrichment analysis of key targets.

**Figure 4:**
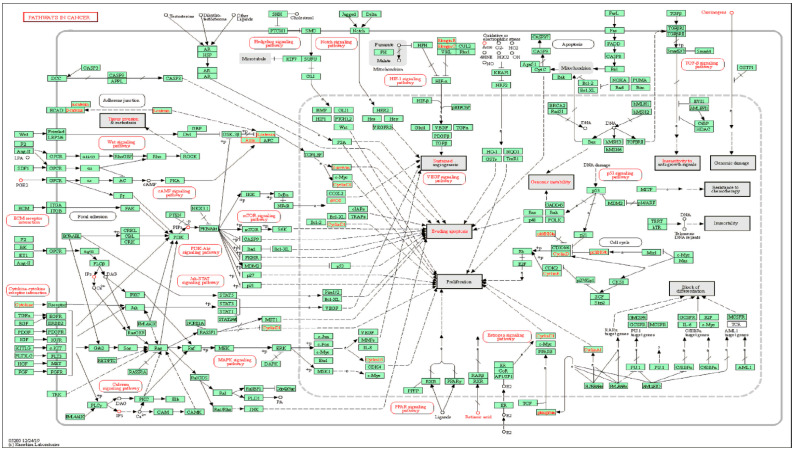
A map showing ‘cancer’-related pathways.

KEGG pathway enrichment analysis, performed using the DAVID database, was undertaken to identify signal pathways associated with the key targets, and we accordingly obtained 58 KEGG pathways, from which we selected the 20 top-ranking pathways based on their *p*-values. In Figure 3, these 20 pathways have been presented in terms of a bubble chart, which notably indicates that the term ‘pathways in cancer’ has the highest gene count and ratio. This term encompasses a range of cellular responses and signaling pathways involved in cancer metabolism (Figure 4). For example, the PI3K-Akt-mTOR signaling pathway is one of the major signaling pathways activated in human cancer and represents a potential therapeutic target for anticancer agents [24,25]. Similarly, the JAK-STAT signaling pathway is a well-established cancer-related signaling pathway, the genetic mutations, amplifications, or polymorphisms of which can cause abnormal activation and promote the development of several cancers [26]. These findings thus provide evidence indicating that the 25 key targets are highly associated with cancer and could thus serve as potential anticancer targets.

#### 2.1.4. Analysis of a Compound-Key Target-Pathway Network

**Figure 5:**
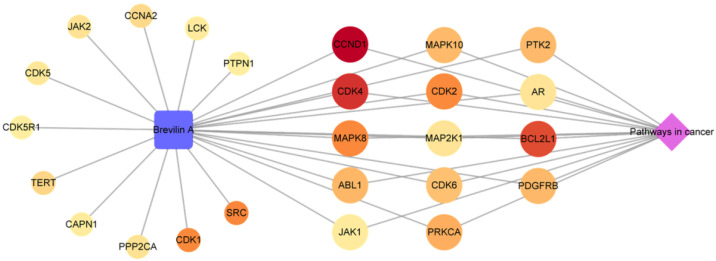
A compound-Key Target-Pathway network for the key targets of brevilin A.

The three categories of compound, key targets, and pathway were merged to establish a C-T-P network consisting of 27 nodes and 39 edges (Figure 5). The blue square, reddish circular, and purple diamond-shaped nodes represent the compounds, key targets, and pathways, respectively. The size and color of the key target nodes indicate the degree and relevance score, respectively. Thus, the larger and redder a node, the higher the degree value and relevance score, indicating that the corresponding node is important in the network. As shown in Figure 5, 14 of the 25 target genes were linked with both the compound and pathway nodes. Among these 14 key targets, CCND1, CDK4, and BCL2L1 showed high relevance scores of 2.873, 2.379, and 2.128, respectively. CCND1 and CDK4 are known as essential cell cycle regulators that promote the G1-S transition in different cell types [27,28]. However, overexpression of these genes is closely associated with tumor growth [29]. BCL2L1 is an apoptosis regulator that encodes both anti-apoptosis (BCL-xL) and pro-apoptosis (BCL-xS) proteins [30]. Overexpression of BCL-xL in cancer cells not only reduces apoptosis but also enhances the migration, invasion, and survival of tumor cells and is associated with acquired chemoresistance [31,32]. This gene is accordingly considered a promising therapeutic target for anticancer agents. Although the relevance score was low according to our results, JAK1 is also known to play a critical role in tumorsphere formation and cell migration [32]. In addition, several studies have revealed that brevilin A is a highly effective JAK inhibitor [33,34]. It showed selective inhibitory activity on JAK-STAT signaling by receding the JAKs activity in cancer cells [33]. Moreover, brevilin A-induced apoptosis and suppressed STAT3 activation by inhibiting phosphorylation of JAK2 [34]. Thus, these findings indicate that brevilin A may exhibit anticancer activity by targeting genes that regulate the cell cycle and metabolism of cancer cells.

### 2.2. The Cytotoxic Effects of Centipeda minima (CMX) and Brevilin A on AGS Human Gastric Cancer Cells

**Figure 6:**
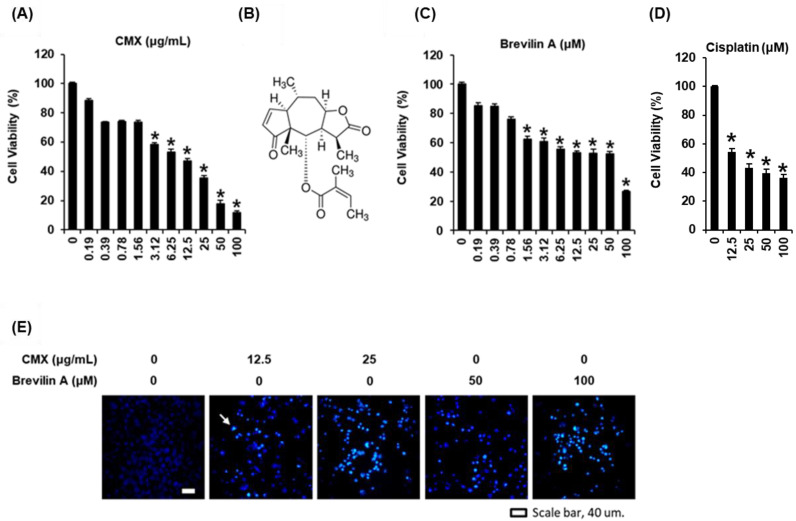
The cytotoxic effects of extracts of *Centipeda minima* (CMX) and brevilin A on AGS human gastric cancer cells. (**A**) The dose-dependent effect of CMX on the viability of AGS cells. (**B**) The chemical structures of brevilin A. The dose-dependent effect of (**C**) brevilin A and (**D**) cisplatin on the viability of AGS cells. (**E**) Hoechst 33342 staining of AGS cells treated with CMX and brevilin A. Brightly stained condensed nuclei (white arrow). Scale bar, 40 µm. The experiments were performed in triplicate. The error bars represent the standard deviation of samples (n = 3). * *p* < 0.05 compared with that of the control.

Although the anticancer effects of CMX and brevilin A have already been reported in various cancer cell lines, the effects of CMX and brevilin A on human gastric cancer cells have not yet been sufficiently well established. Thus, in our study, we applied a network pharmacology approach to elucidate the effect on human gastric cancer cells based on potential targets and mechanisms related to the predicted anticancer activity of brevilin A. In our study, we used an AGS human gastric cancer cell line as an in vitro model to examine the anticancer therapeutic potential of these two products. We accordingly found that both CMX and brevilin A reduced the viability of AGS cells, with CMX being found to have a 24-h half-inhibitory concentration (IC50) of 9.73 ± 1.29 µg/mL (Figure 6A). Comparatively, previous studies using the human breast cancer cell lines MCF-7 and MDA-MB-231 and the human nasopharyngeal carcinoma cell line CNE-1 have reported 24-h IC50 values for CMX of 27.17, 19.96, and 41.57 ± 0.17 µg/mL in MCF-7, MDA-MB-231, and CNE-1 cells, respectively [1,2].

In response to the treatment of AGS cells with CMX-derived brevilin A at concentrations up to 100 μM, we observed a concentration-dependent reduction in cell viability, with a 24-h IC50 value of 54.69 ± 1.38 μM (Figure 6B,C). At the same concentration (100 μM), brevilin A had a better toxic effect than cisplatin, known for curing various types of cancer (Figure 6D). Previous studies have reported that brevilin A shows anticancer effects against a range of cancer cells, including a human lung adenocarcinoma cell line (A549), human breast cancer cell lines (MCF-7 and MDA-MB-231), a human colon cancer cell line (SW480), human liver cancer cell lines (HepG2 and SMMC-7221), and human melanoma cell lines (A375 and A2058), with respective 24-h IC50 values of 10, 12, 7, 13, 13, 17, 1.98, and 2.71 μM [5,6,7]. 

To further confirm the mechanisms associated with the reduced viability of AGS cells in response to treatment with CMX and brevilin A, we stained cells with Hoechst 33342 dye (blue), which binds to nucleic acids. As shown in Figure 6E, we detected bright blue (white arrow) staining of condensed nuclear material, the intensity of which increased with an increase in the concentrations of CMX (12.5 and 25 µg/mL) and brevilin A (50 and 100 µM). Thus, we established that CMX and brevilin A induce changes in the nuclear morphologies in AGS cells, which in turn indicates that these extracts may be effective cytotoxic agents. Given that apoptotic cell death is characterized by nuclear condensation [33], we subsequently evaluated whether CMX and brevilin A can contribute to the upregulated expression of apoptosis-related proteins.

### 2.3. The Effects of Centipeda minima (CMX) and Brevilin A on the Expression of Apoptosis-Related Proteins in AGS Human Gastric Cancer Cells

**Figure 7:**
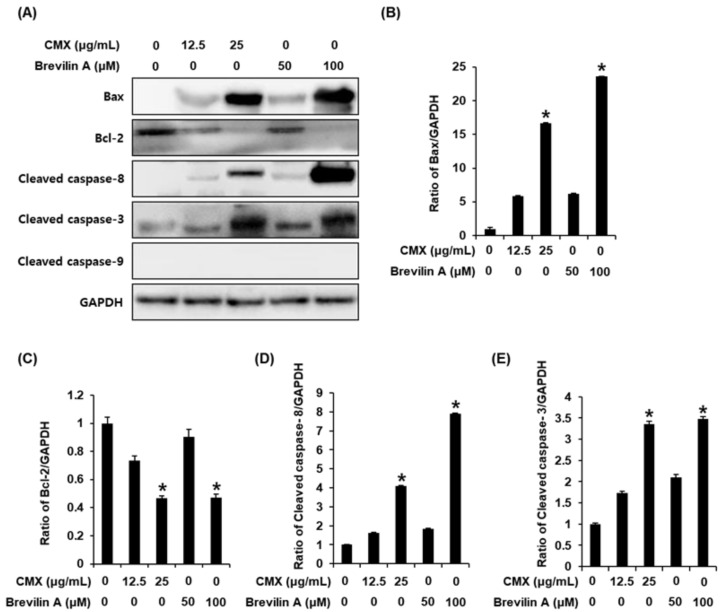
The effects of Centipeda minima (CMX) and brevilin A on the expression of apoptosis-related proteins in AGS human gastric cancer cells. (**A**) Representative images of western blotting for Bax, Bcl-2, cleaved caspase-8, cleaved caspase-3, cleaved caspase-9, and GAPDH in AGS cells treated with CMX and brevilin A for 24 h. (**B**–**E**) Bar graphs presenting the densitometric quantification of the respective western blot bands. The experiment was performed in triplicate. The error bars represent the standard deviation of samples (n = 3). * *p* < 0.05 compared with that of the control.

In response to changes in the extracellular environment induced by anticancer drugs, cells initiate a death receptor-activated extrinsic pathway [34], which is mediated by the activation of initiator caspase-8 that proteolytically activates the cleavage of Bid to bind to either the pro-apoptotic Bax protein or anti-apoptotic Bcl-2 protein [35]. The subsequent intrinsic apoptosis pathway commences within the cancer cells with the release of mitochondrial cytochrome c, after which procaspase-9 cleavage initiates apoptotic cell death by cleaving effector caspase-3 [36]. A previous study using MDA-MB-231 cells has shown that treatment of these cells with CMX (20 μg/mL) promotes the upregulated cleavage of caspase-9 and -3, although it had no similar effect on the protein levels of cleaved caspase-8 [1]. In a further study, CMX (25 μg/mL) was observed to induce apoptosis by upregulating the expression of cleaved caspase-8, cleaved caspase-9, and cleaved caspase-3 while downregulating the Bax/Bcl-2 ratio [2], whereas brevilin A (10 μM) has been found to upregulate the expression of cleaved caspase-3 in A549, MCF-7, MDA-MB-231, and SW480 cells [5]. Based on these observations, we thus performed western blotting to determine whether the apoptosis of AGS cells is induced via an extrinsic or intrinsic apoptotic pathway in the presence of CMX (12.5 and 25 μg/mL) and brevilin A (50 and 100 μM). Interestingly, we found that the protein levels of cleaved caspase-8 were increased by both CMX and brevilin A in a concentration-dependent manner, whereas these extracts appeared to have no significant effect on the levels of cleaved caspase-9 protein. These findings would thus appear to indicate that CMX and brevilin A do not act by inducing an intrinsic apoptosis pathway. In addition, we observed higher levels of the pro-apoptotic protein Bax in response to treatment with CMX and brevilin A, whereas there were corresponding concentration-dependent reductions in the levels of the anti-apoptotic protein Bcl-2 (Figure 7). These findings were consistent with those obtained in our network pharmacology analysis. In the extrinsic apoptosis pathway, activated caspase-8 induces apoptotic cell death by cleaving effector caspase-3 [37], and in the present study, we found that treatment of AGS cells with CMX and brevilin A enhanced the expression of cleaved caspase-3 (Figure 7). These findings thus tend to indicate that CMX and brevilin A induce an extrinsic apoptosis pathway in AGS cells associated with the activation of caspase-8 and -3. This putative role of caspase-8 in the apoptosis in AGS cells following treatment with CMX and brevilin A was confirmed by annexin V staining.

On the other hand, we also examined the protein levels of cyclin D1 and CDK4 as cell cycle regulators that control the G1 to S phase of the cell cycle. However, CMX and brevilin A did not affect the protein levels of cyclin D1 and CDK4 (data not shown). This result indicated that CMX and brevilin A did not contribute to the cell cycle arrest.

### 2.4. The Effects of Centipeda minima (CMX) and Brevilin A on Apoptotic Cell Death in AGS Human Gastric Cancer Cells

**Figure 8:**
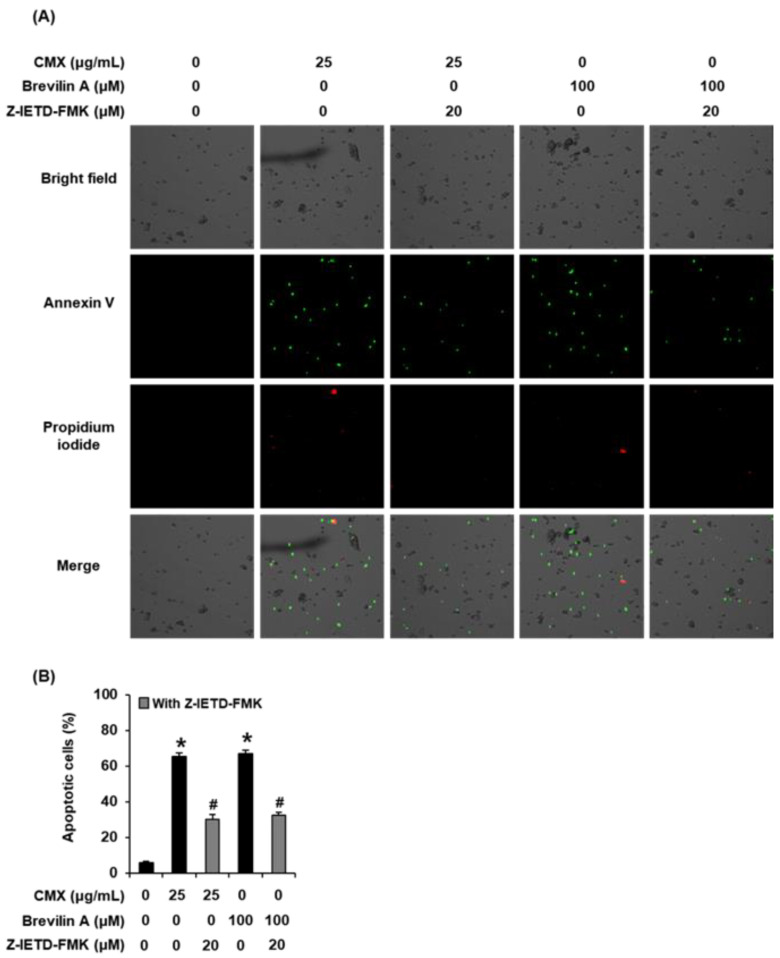
The effects of *Centipeda minima* (CMX), brevilin A, and Z-IETD-FMK (a caspase-8 inhibitor) on apoptotic cell death in AGS human gastric cancer cells. (**A**) Representative images of apoptotic cells stained with annexin V (green) and propidium iodide (red) were obtained using a Tali Image-Based Cytometer. (**B**) The experiment was performed in triplicate. The error bars represent the standard deviation of samples (n = 3). * *p* < 0.05 compared with that of the control. # *p* < 0.05 difference with Z-IETD-FMK group.

As shown in Figure 8, treatment with CMX (25 μg/mL) and brevilin A (100 μM) promoted increases in the percentages of annexin V-positive cells, whereas, in contrast, 20 µM Z-IETD-FMK (a caspase-8 inhibitor) was found to cause a reduction in the percentages of annexin V-positive cells. These findings indicate that the anticancer activities of CMX and brevilin A may be mediated via upregulation of caspase-8. 

Collectively, the results obtained in this study provide evidence to indicate that CMX and brevilin A are potentially promising candidates for further development as herbal medicines for the treatment of gastric cancer. We will determine whether CMX and brevilin A induce or inhibit autophagy as another type of cell death in AGS cells. We will also evaluate in future plans the anticancer activities of CMX and brevilin A in Balb/c mice bearing AGS xenografts.

## 3. Materials and Methods

### 3.1. Extraction and Isolation

*C. minima* (whole plants including flowers, stems, leaves, and roots) was purchased in December 2019 from Natural-herb (Goesan, Korea). One of the authors (J.P.) identified the material. A voucher specimen of the material (CM-2019-001) was deposited in the herbarium at Kyungsung University. CMX was prepared by D. Nature Co., Ltd. (Seongnam, Korea), inducing phase separation in the emulsion to efficiently separate brevilin A from *C. minima* as reported previously [11]. The amount of brevilin A in *C. minim* was 0.29% [11]. 

### 3.2. Network Pharmacology Analysis

#### 3.2.1. Acquisition of the Predicted and Disease-Related Targets of Brevilin A

A SMILES code of brevilin A was obtained from the PubChem database [https://pubchem.ncbi.nlm.nih.gov/ (accessed on 18 August 2021)] and entered into the SwissTargetPrediction database [http://www.swisstargetprediction.ch/ (accessed on 18 August 2021)] [38] to determine the predicted targets of brevilin A. Using “Anticancer” as a keyword; we searched the GeneCards database [https://www.genecards.org/ (accessed on 18 August 2021)] to identify disease-related targets.

#### 3.2.2. Acquisition of Potential Targets

As potential targets, we selected common targets among those predicted for brevilin A and anticancer-related targets, which were visualized by generating a Venn diagram using Venny 2.1 [https://bioinfogp.cnb.csic.es/tools/venny/index.html (accessed on 18 August 2021)] [39], whereas the DisGeNET database [https://www.disgenet.org/search (accessed on 18 August 2021)] [40] was used to retrieve information regarding the specific protein classes of the potential targets.

#### 3.2.3. Construction and Analysis of a Protein–Protein Interaction Network

Protein–protein interactions (PPIs) of the potential targets were analyzed using the STRING database [https://string-db.org/ (accessed on 18 August 2021)] [41]. The analysis parameters were set at a high confidence of 0.700 and a medium false discovery rate (FDR) stringency of 5%. The resultant data were imported into Cytoscape software (Ver. 3.9.0) [42] to construct and analyze the PPI network of the potential targets. The three parameters “degree”, “betweenness centrality”, and “closeness centrality” were used to estimate the topological features of network nodes, based on which key targets were selected from among the potential targets.

#### 3.2.4. Kyoto Encyclopedia of Genes and Genomes Pathway Enrichment Analysis

Kyoto Encyclopedia of Genes and Genomes (KEGG) pathway enrichment analysis of the key targets was performed using the DAVID Bioinformatics Resources 6.8 database [https://david.ncifcrf.gov/home.jsp (accessed on 18 August 2021)] [43]. The FDR error control method (FDR < 0.05) was used to correct the *p*-value and a *p*-value < 0.05 was set as a threshold value to identify significant signaling pathways. The results of the analysis were visualized using ImageGP [http://www.ehbio.com/ImageGP (accessed on 18 August 2021)].

#### 3.2.5. Construction and Analysis of a Compound-Key Target-Pathway (C-T-P) Network

An integrated network of compounds, key targets, and pathways was constructed and analyzed using Cytoscape software (Ver. 3.9.0, Seattle, WA, USA).

### 3.3. Cell Culture

Cultures of the AGS human gastric adenocarcinoma cell line (CRL-1739, American Type Culture Collection, Manassas, VA, USA) were routinely maintained in Roswell Park Memorial Institute 1640 medium (RPMI 1640; Cellgro, Manassas, VA, USA) supplemented with 100 units/mL penicillin, 10% fetal bovine serum, and 100 mg/mL streptomycin (all purchased from Sigma-Aldrich, St. Louis, MO, USA) in a humidified atmosphere containing 5% CO_2_ at 37 °C.

### 3.4. Ez-Cytox Assay

The viability of AGS cells was determined using an Ez-Cytox assay reagent kit (Dail Lab Service Co., Seoul, Korea). Using 96-well plates, cells in RPMI 1640 medium were treated with CMX (0.19, 0.39, 0.78, 1.56, 3.12, 6.25, 12.5, 25, 50, and 100 μg/mL) and brevilin A (0.19, 0.39, 0.78, 1.56, 3.12, 6.25, 12.5, 25, 50, and 100 μM) for 24 h at the end of which, 10% (*v/v*) Ez-Cytox assay reagent was added to each well, and the optical density of well contents was recorded at 450 nm wavelength using a PowerWave XS microplate reader (Bio-Tek Instruments, Winooski, VT, USA). 

### 3.5. Cell Staining Using Hoechst 33342

AGS cells were grown in six-well plates and treated with CMX (25 μg/mL) or brevilin A (100 μM) for 24 h, after which, the cells were stained with Hoechst 33342 solution (Sigma-Aldrich, St. Louis, MO, USA) for 10 min and observed under a fluorescence microscope to assess nuclear condensation.

### 3.6. Western Blot Analysis

AGS cells were treated with CMX (12.5 and 25 μg/mL) or brevilin A (50 and 100 μM) as described in the preceding section. Having incubated for 24 h, the treated AGS cells were harvested and lysed with ice-cold RIPA-lysis buffer (Upstate Biotechnology, Lake Placid, NY, USA) for 20 min on ice. Aliquots (20 µg) of the resulting whole-cell extracts were resolved electrophoretically on 10% SDS-PAGE gels, and the resolved protein bands were subsequently transferred to a nitrocellulose membrane. The membranes were thereafter probed with epitope-specific primary antibodies (Cell Signaling Technology, Inc., Danvers, MA, USA) against the following proteins: Bcl-2 (# 4223S), Bax (# 5023S), cleaved caspase-8 (# 9496S), cleaved caspase-9 (# 20750S), cleaved caspase-3 (# 9661S), and glyceraldehyde-3-phosphate dehydrogenase (GAPDH) (# 5174S). Horseradish peroxidase-conjugated rabbit antibodies (Cell Signaling Technology, Inc., Danvers, MA, USA) were used as a secondary antibody. Bound antibodies were subsequently visualized using enhanced chemiluminescence Advance Western Blotting Detection Reagents (GE Healthcare, Chicago, IL, USA) and detected using a FUSION Solo Chemiluminescence System (PEQLAB Biotechnologie GmbH, Erlangen, Germany).

### 3.7. Cell Staining with Annexin V and Propidium Iodide

AGS cells were treated with CMX (25 μg/mL), brevilin A (100 μM), or Z-IETD-FMK (20 μM) as described in the preceding sections. Z-IETD-FMK (purchased from Abcam, Cambridge, MA, USA) was used as a specific inhibitor of the initiator caspase-8. Treated cells were harvested after 24 h and washed with annexin binding buffer (Invitrogen; Temecula, CA, USA). The staining was performed by the addition of annexin V, Alexa Fluor 488, or propidium iodide (Invitrogen; Temecula, CA, USA), followed by incubation in the dark at room temperature for 30 min. The percentage of apoptotic cells was calculated automatically using a Tali Image-Based Cytometer (Invitrogen).

### 3.8. Statistical Analysis

All assays were performed in triplicate and repeated at least three times, and all data are presented as the means ± standard deviation. Statistical significance was determined using a one-way analysis of variance and multiple comparisons with Bonferroni correction. A *p*-value < 0.05 was taken to be indicative of statistical significance. All analyses were performed using SPSS Statistics ver. 19.0 statistical software (SPSS Inc., Chicago, IL, USA).

## 4. Conclusions

In this study, we deduced the potential targets and underlying mechanisms of the anticancer activities of brevilin A using a network pharmacology approach. CCND1, CDK4, and BCL2L1 were identified as the key anticancer-associated genes targeted by brevilin A, and we identified a number of pathways, including the PI3K-Akt-mTOR, JAK-STAT, and MAPK signaling pathways, which are predicted to be involved in the anticancer activities of brevilin A. Both CMX and brevilin A were found to reduce the viability of AGS cells and shown to induce apoptosis by promoting the upregulated expression of cleaved caspase-8 and cleaved caspase-3 and reducing the Bax/Bcl-2 ratio. These findings are partially consistent with those obtained based on our network pharmacology analysis and provide evidence indicating that CMX and brevilin A may have potential utility as novel sources of herbal medicine for the treatment of gastric cancer.

## Figures and Tables

**Table 1:** A list of potential brevilin A predicted/anticancer-related targets.

No.	UniProt ID	Gene	Targets	Protein Class
1	P08183	*ABCB1*	ATP binding cassette subfamily B member 1	Transporter
2	P09874	*PARP1*	poly (ADP-ribose) polymerase 1	-
3	P24385	*CCND1*	cyclin D1	Enzyme modulator
4	P11802	*CDK4*	cyclin-dependent kinase 4	Kinase
5	Q07817	*BCL2L1*	BCL2 like 1	Signaling
6	P35354	*PTGS2*	prostaglandin-endoperoxide synthase 2	Enzyme
7	P12931	*SRC*	SRC proto-oncogene, non-receptor tyrosine kinase	Kinase
8	P45983	*MAPK8*	mitogen-activated protein kinase 8	Kinase
9	P06493	*CDK1*	cyclin-dependent kinase 1	Kinase
10	P24941	*CDK2*	cyclin-dependent kinase 2	Kinase
11	P09917	*ALOX5*	arachidonate 5-lipoxygenase	Enzyme
12	P11511	*CYP19A1*	cytochrome P450 family 19 subfamily A member 1	Enzyme
13	P52333	*JAK3*	Janus kinase 3	Kinase
14	P17252	*PRKCA*	protein kinase C alpha	Kinase
15	O14920	*IKBKB*	inhibitor of nuclear factor-kappa B kinase subunit beta	Kinase
16	P14635	*CCNB1*	cyclin B1	Enzyme modulator
17	P00519	*ABL1*	ABL proto-oncogene 1, non-receptor tyrosine kinase	Kinase
18	P09619	*PDGFRB*	platelet-derived growth factor receptor beta	Kinase
19	P53779	*MAPK10*	mitogen-activated protein kinase 10	Kinase
20	Q05397	*PTK2*	protein tyrosine kinase 2	Kinase
21	P37231	*PPARG*	peroxisome proliferator-activated receptor gamma	Nuclear receptor
22	Q07869	*PPARA*	peroxisome proliferator-activated receptor alpha	Nuclear receptor
23	P07858	*CTSB*	cathepsin B	Enzyme
24	Q00534	*CDK6*	cyclin-dependent kinase 6	Kinase
25	Q9NR96	*TLR9*	toll-like receptor 9	-
26	P17948	*FLT1*	fms-related receptor tyrosine kinase 1	Kinase
27	P04150	*NR3C1*	nuclear receptor subfamily 3 group C member 1	Nuclear receptor
28	P05129	*PRKCG*	protein kinase C gamma	Kinase
29	P30305	*CDC25B*	cell division cycle 25B	Enzyme
30	O14746	*TERT*	telomerase reverse transcriptase	Enzyme
31	P20248	*CCNA2*	cyclin A2	Enzyme modulator
32	P34913	*EPHX2*	epoxide hydrolase 2	Enzyme
33	P67775	*PPP2CA*	protein phosphatase 2 catalytic subunit alpha	-
34	O60674	*JAK2*	Janus kinase 2	Kinase
35	O60885	*BRD4*	bromodomain containing 4	Epigenetic regulator
36	Q02750	*MAP2K1*	mitogen-activated protein kinase kinase 1	Kinase
37	P10275	*AR*	androgen receptor	Nuclear receptor
38	P11362	*FGFR1*	fibroblast growth factor receptor 1	Kinase
39	Q00535	*CDK5*	cyclin-dependent kinase 5	Kinase
40	P03956	*MMP1*	matrix metallopeptidase 1	Enzyme
41	P08254	*MMP3*	matrix metallopeptidase 3	Enzyme
42	Q96RI1	*NR1H4*	nuclear receptor subfamily 1 group H member 4	Nuclear receptor
43	Q99572	*P2RX7*	purinergic receptor P2X 7	Ion channel
44	P49356	*FNTB*	farnesyltransferase, CAAX box, beta	-
45	P30536	*TSPO*	translocator protein	-
46	P23458	*JAK1*	Janus kinase 1	Kinase
47	O75874	*IDH1*	isocitrate dehydrogenase (NADP(+)) 1	-
48	P06239	*LCK*	LCK proto-oncogene, SRC family tyrosine kinase	Kinase
49	P07333	*CSF1R*	colony-stimulating factor 1 receptor	Kinase
50	Q13627	*DYRK1A*	dual-specificity tyrosine phosphorylation regulated kinase 1A	Kinase
51	P07384	*CAPN1*	calpain 1	Enzyme
52	Q9UDY8	*MALT1*	MALT1 paracaspase	Enzyme
53	P48039	*MTNR1A*	melatonin receptor 1A	G-protein-coupled receptor
54	P49354	*FNTA*	farnesyltransferase, CAAX box, alpha	Enzyme
55	Q12884	*FAP*	fibroblast activation protein alpha	Enzyme
56	P18031	*PTPN1*	protein tyrosine phosphatase non-receptor type 1	-
57	Q15078	*CDK5R1*	cyclin-dependent kinase 5 regulatory subunit 1	Enzyme modulator
58	P25774	*CTSS*	cathepsin S	Enzyme
59	P22001	*KCNA3*	potassium voltage-gated channel subfamily A member 3	Ion channel

**Table 2:** A list of key targets based on protein–protein interaction network topological analysis.

No.	UniProt ID	Gene	Degree	Relevance Score	Betweenness Centrality	Closeness Centrality
1	P12931	*SRC*	18	1.557	0.216	0.800
2	P00519	*ABL1*	12	0.882	0.085	0.667
3	P06493	*CDK1*	11	1.528	0.034	0.649
4	P24385	*CCND1*	11	2.873	0.024	0.649
5	P24941	*CDK2*	10	1.517	0.037	0.600
6	Q00534	*CDK6*	10	0.748	0.018	0.632
7	P11802	*CDK4*	10	2.379	0.022	0.632
8	Q00535	*CDK5*	10	0.196	0.039	0.615
9	O60674	*JAK2*	10	0.259	0.046	0.615
10	P20248	*CCNA2*	9	0.392	0.016	0.571
11	Q02750	*MAP2K1*	9	0.240	0.058	0.600
12	P06239	*LCK*	9	0.139	0.027	0.615
13	P45983	*MAPK8*	8	1.536	0.026	0.600
14	P09619	*PDGFRB*	7	0.868	0.009	0.571
15	Q07817	*BCL2L1*	7	2.128	0.018	0.585
16	P18031	*PTPN1*	6	0.098	0.012	0.571
17	Q05397	*PTK2*	6	0.854	0.014	0.545
18	P23458	*JAK1*	6	0.139	0.009	0.522
19	P67775	*PPP2CA*	6	0.277	0.016	0.571
20	P10275	*AR*	5	0.240	0.003	0.533
21	P07384	*CAPN1*	5	0.139	0.021	0.545
22	O14746	*TERT*	5	0.439	0.011	0.500
23	Q15078	*CDK5R1*	4	0.098	0.005	0.500
24	P17252	*PRKCA*	4	1.007	0.011	0.511
25	P53779	*MAPK10*	4	0.854	0.001	0.522
